# Increased high sensitivity C-reactive protein in more severe wheeze/asthma phenotypes in child- and adulthood in ALLIANCE

**DOI:** 10.1186/s12931-026-03840-x

**Published:** 2026-07-27

**Authors:** Lena Lagally, Lena Ullemeyer, Jimmy Omony, Kristina Gottschau, Francesco Foppiano, Mustafa Abdo, Vera Veith, Clemens Thölken, Alexander Hose, Nicole Maison, Ruth Grychtol, Anna-Maria Dittrich, Lennart Riemann, Markus Weckmann, Inke König, Lea Kronziel, Sabina Illi, Silke van Koningsbruggen-Rietschel, Klaus F. Rabe, Harald Renz, Gesine Hansen, Folke Brinkmann, Matthias V. Kopp, Erika von Mutius, Thomas Bahmer, Markus Ege, Chrysanthi Skevaki, Bianca Schaub, ALLIANCE Study Group, Mira Bürk, Sybille Contento, Silvia Gschwendtner, Constanze Jakwerth, Michael Schloter, Carsten Schmidt-Weber, Esther Zeitlmann, Heike Biller, Constantin Blanke-Roeser, Karoline I. Gaede, Wiebke Hagedorn, Nikolas Jacobs, Anne-Marie Kirsten, Gyde Nissen, Wasifa Nurieva, Frauke Pedersen, Loana Penner, Isabell Ricklefs, Silke Szymczak, Benjamin Waschki, Henrik Watz, Christopher Wirks, Marie Bickes, Mifflin-Rae Calvero, David S. DeLuca, Svenja Gaedcke, Anika Habener, Christine Happle, Adan Chari Jirmo, Bin Liu, Svenja Foth, Inga Jerrentrup, Stefanie Weber, Miguel A. Alejandre Alcazar, Lena Keufken, Ernst Rietschel, Tobias Trojan, Jan-Christoph Thomassen

**Affiliations:** 1 Department of Paediatric Allergology, Dr. von Hauner Children’s Hospital, LMU University Hospital, LMU Medizin, Ludwig-Maximilians-Universität München, Munich, Germany; 2 Comprehensive Pneumology Center, Munich (CPC-M), German Center for Lung Research (DZL), Munich, Germany; 3 Institute for Medical Information Processing, Biometry, and Epidemiology (IBE), LMU Medizin, Ludwig-Maximilians-Universität München, Pettenkofer School of Public Health, Munich, Germany; 4 Institute for Asthma and Allergy Prevention (IAP), Helmholtz Zentrum Munich, German Research Center for Environmental Health (GmbH), Neuherberg, Germany; 5 LungenClinic Grosshansdorf GmbH, Grosshansdorf, Germany; 6 Airway Research Center North (ARCN), German Center for Lung Research (DZL), Grosshansdorf, Germany; 7 Department of Pneumology and Critical Care Medicine, Thoraxklinik at Heidelberg University Hospital, Translational Lung Research Center Heidelberg, Heidelberg, Germany; 8 Center for Synthetic Microbiology (SYNMIKRO), Philipps-Universität Marburg, Marburg, Germany; 9 Department of Paediatric Pneumology, Allergology and Neonatology, Hannover Medical School, Hannover, Germany; 10 Biomedical Research in Endstage and Obstructive Lung Disease Hannover (BREATH), German Center for Lung Research (DZL), Hannover, Germany; 11 Cluster of Excellence RESIST (EXC 2155), Hannover Medical School, Hannover, Germany; 12 Institute of Immunology, Hannover Medical School, Hannover, Germany; 13 University Children’s Hospital, Luebeck, Germany; 14 Airway Research Center North (ARCN), German Center for Lung Research (DZL), Luebeck, Germany; 15 Division of Epigenetics in Chronic Lung Disease, Priority Area Chronic Lung Diseases, Leibniz Lung Center, Research Center Borstel, Borstel, Germany; 16 Institute for Medical Biometry and Statistics, University Luebeck, University Medical Centre Schleswig-Holstein, Campus Luebeck, Luebeck, Germany; 17 Department of Pediatrics, Faculty of Medicine and University Hospital Cologne, University of Cologne, Cologne, Germany; 18 Institute of Laboratory Medicine and Pathobiochemistry, Molecular Diagnostics, University of Marburg, Marburg, Germany; 19 University of Gießen and Marburg Lung Center (UGMLC), German Center for Lung Research (DZL), Gießen, Germany; 20 Department of Paediatric Respiratory Medicine, Inselspital, University Children’s Hospital of Bern, University of Bern, Bern, Switzerland; 21 Department of Environmental Health, Harvard T.H. Chan School of Public Health, Boston, USA; 22 Centre for Research and Education in Public Health (CEPHRE), Academy of Athens, Athens, Greece; 23 German Center for Child and Adolescent Health (DZKJ), Dr. von Hauner Children’s Hospital, LMU University Hospital, LMU Medizin, Ludwig-Maximilians-Universität München, Munich, Germany

**Keywords:** Asthma, Wheeze, Asthma treatment, Clinical immunology, Epidemiology, Inflammation, Pediatrics

## Abstract

**Background:**

The relevance of high-sensitivity C-reactive protein (hsCRP), a marker of low-grade systemic inflammation, remains unclear with regard to its association with severity and clinical outcomes in wheeze/asthma. We aimed to assess the role of hsCRP across different phenotypes and severity levels.

**Methods:**

We studied children with preschool wheeze (≥ 2 episodes), and patients with GINA-defined asthma (school-age/adult) compared with healthy controls (HCs) in the well-characterized ALLIANCE (All Age Asthma Cohort) study. HsCRP was measured (AU5800®-CRP-Latex test) in 944 study participants (pediatric: *n* = 728; adult: *n* = 216) at baseline. Age-stratified analyses (age groups 0–5, 6–18, ≥ 18 years) of standardized log_10_-transformed hsCRP concentrations (age, sex, BMI, site) were performed using univariable tests and regression models. The validated ASSESS score and its dimensions (exacerbations, lung function, inhaled corticosteroids, symptom control) were primary outcomes.

**Results:**

Adult patients with asthma showed higher hsCRP than HCs (OR 2.22, 95% CI 1.56–3.24). Across all ages, hsCRP increased with clinical severity of wheeze/asthma. The ASSESS score correlated positively with hsCRP in patients aged ≥ 6 years (*R* = 0.19, *p* = 0.007*).* hsCRP was increased in school-age asthmatics with prior exacerbations (OR 1.37, 95% CI 1.01–1.87), and in adult asthmatics with impaired lung function (*R* = 0.2, *p* = 0.013). Inhaled corticosteroid use was associated with lower hsCRP in preschool wheezers (OR 0.66, 95% CI 0.50–0.85) but higher levels in adults (OR 1.99, 95% CI 1.02–4.09).

**Conclusions:**

HsCRP was increased in adult asthmatics compared to HCs and was associated with several severity-related clinical characteristics. ICS use was associated with higher hsCRP levels in adults, potentially reflecting greater disease severity, whereas ICS use in preschool wheezers was associated with lower hsCRP levels. These age-dependent effects may mirror varying disease courses across the lifespan and progression of asthma. The association of hsCRP with asthma severity in child- and adulthood may indicate its potential relevance for the course of disease and monitoring clinical outcomes. Future longitudinal studies are needed to assess, whether hsCRP may support therapy monitoring.

**Trial registration:**

ClinicalTrials.gov; Pediatric arm: NCT02496468, Registration date: 03 July 2015; Adult arm: NCT02419274, Registration date: 14 April 2015.

**Supplementary Information:**

The online version contains supplementary material available at 10.1186/s12931-026-03840-x.

## Introduction

Asthma is a heterogeneous chronic respiratory disease affecting approximately 300 million people worldwide. Its prevalence is estimated to be around 10% in the global pediatric population, making it one of the most common chronic conditions in childhood [1, 2]. Through airway inflammation, bronchial hyperreactivity, and episodic airway obstruction, asthma can present with symptoms such as cough, shortness of breath, and chest tightness, along with recurrent airway obstruction, which can reduce overall quality of life and affect daily activities [3].

Asthma comprises different phenotypes, each associated with varying degrees of severity and clinical course. The multidimensional Asthma Severity Scoring System (ASSESS) [4], developed to reflect asthma severity across distinct dimensions, was validated for children [5]. It comprises factors such as asthma control, quantified by the Asthma Control Test [6] (ACT), lung function (FEV_1_), asthma medication use, and exacerbations.

Accurate diagnosis and disease management [7, 8] as well as pharmacological interventions such as inhaled corticosteroids and bronchodilators [9, 10] are crucial for symptom control. Biomarkers can facilitate risk and treatment stratification [11, 12], accounting for inter-individual susceptibility to adverse outcomes related to asthma symptom control. High-sensitivity C-reactive protein (hsCRP) levels have been suggested to reflect asthma severity by indicating low-grade systemic inflammation [13].

CRP is a well-established biomarker of inflammation, first identified as an acute-phase reactant produced by the liver. However, standard CRP assays, with detection limits typically in the range of 3–8 mg/L, lack the sensitivity to measure low-grade chronic inflammation. hsCRP assays can detect CRP levels as low as 0.04 mg/L, improving the ability to monitor subtle inflammatory processes [14, 15]. HsCRP is associated with several chronic diseases. Studies have shown that it contributes significantly to improved cardiovascular risk assessment by predicting coronary artery disease through low-grade inflammation [16, 17]. HsCRP is also a marker of diseases such as diabetes mellitus [18] and chronic obstructive pulmonary disease (COPD) [19, 20].

Disease activity in asthma has been associated with increased hsCRP levels [21, 22] in children and adults with asthma and higher disease severity. Inflammatory mechanisms driving asthma exacerbations are reflected particularly by elevated hsCRP levels [23, 24]. Increased hsCRP levels are also linked to obesity – a comorbidity associated with asthma development and severity [25, 26]. However, most studies were underpowered, had narrow age ranges, and limited phenotyping. Studies with large asthma case numbers, a wide age range from childhood to adulthood, and comprehensive documentation of various clinical characteristics are still lacking. Given the developmental differences in inflammatory responses across age, analyses stratified by pediatric and adult age groups are essential to improve the interpretability of hsCRP in wheeze and asthma.

This study aimed to investigate the role of hsCRP in asthma severity outcomes and individual patient characteristics. Therefore, we first compared the hsCRP levels of patients with wheeze and asthma with those of healthy individuals. Second, we assessed how low-grade systemic inflammation, reflected by hsCRP, relates to distinct ASSESS dimensions – including symptom control, lung function, medication use, and exacerbations – in pediatric preschool wheezers and school-age and adult patients with asthma.

## Methods

### Study design and cohort structure

Eligible study participants were recruited as part of the multicenter, prospective All Age Asthma Cohort [27–29] (ALLIANCE), initiated by the German Center of Lung Research (DZL). The cohort comprises pediatric and adult patients as well as healthy controls (HCs) from seven hospitals across Germany. The pediatric study arm included young children aged 6 months to 5 years with ≥ 2 wheezing episodes within the past 12 months as reported by their parents (“preschool wheezers”). Children aged 6–18 years and adults aged ≥ 18 years with a physician-diagnosed history of asthma, defined according to the Global Initiative for Asthma (GINA) [10] and German national asthma guidelines [30], were included as “school-age patients with asthma” and “adult patients with”. Current or former smoking was not an exclusion criterion. Healthy pediatric and adult controls were recruited at the same centers if they had no history of asthma or preschool wheeze, irrespective of other allergic conditions.

### Ethics approval and consent

The study was approved by local ethics committees (lead: University of Luebeck Ethics Committee; reference no. AZ 12–215) and conducted in accordance with the Declaration of Helsinki. Informed consent was obtained from parents or legal representatives of children < 8 years, and assent was obtained from children ≥ 8 years. All adult participants provided written informed consent before enrollment. Detailed descriptions of the ALLIANCE study protocol, recruitment, and inclusion/exclusion criteria have been published previously [27]. The study is registered at clinicaltrials.gov (pediatric arm: NCT02496468; adult arm: NCT02419274).

### Study population for hsCRP analyses

Pediatric (*n* = 784) and adult participants (*n* = 254) with available serum hsCRP measurements at baseline visits were included in the present analysis. Observations were excluded if hsCRP levels were ≥ 10 mg/L, patient body temperature was ≥ 38.0 °C, the neutrophil-lymphocytes ratio was > 9 [31, 32], or BMI data were missing (Supplementary Figure S1). In addition, patients aged ≥ 6 years with an ASSESS score of zero or missing ASSESS score were excluded from analyses as misclassification could not be ruled out (Supplementary Figure S1).

hsCRP concentrations were measured centrally at the Institute of Laboratory Medicine, Philipps University Marburg, using the CRP-Latex test on the AU5800® System (Beckman Coulter) CRP-Latex test. The analytical range for hsCRP was 0.08–10 mg/L. Measurements below the lower limit of quantification (< 0.08 mg/L; in 8.16% of all samples; age 0–5 years: *n* = 30; age 6–18 years: *n* = 46; age ≥ 18 years: *n* = 1) were set to 0.07 mg/L for inclusion in the analyses. This pragmatic substitution retained observations with very low hsCRP concentrations, avoided assigning a value of zero before log-transformation, and preserved their position below the assay detection threshold.

### Assessment of disease severity

Disease severity in pediatric and adult asthmatics was quantified using the ASSESS score, computed as described previously (Supplementary Table S1) [5]. To study the distinct components of ASSESS, ACT scores (≥ 4 years), FEV_1_ z-scores (≥ 6 years), inhaled corticosteroids (ICS) and/or long-acting β agonists (LABA/ICS) use within the past 12 months, and exacerbations were evaluated. Exacerbations within the previous 12 months were defined based on oral corticosteroid (OCS) use and/or hospitalisation due to respiratory symptoms. In adults, exacerbations were defined as symptom worsening requiring ≥ 3 consecutive days of OCS treatment or ongoing OCS therapy, whereas in children, any OCS-treated episode qualified as an exacerbation.

In children (≤ 18 years), exacerbations (according to GINA [10]) were additionally documented for the 4 weeks preceding the study visit. We also had information about acute ear, nose and/or throat (ENT) symptoms at the study visit, indicating a preceding, but not acute infection, as body temperature was < 38 °C. Detailed definitions of other variables are provided in the Supplementary Methods.

### Statistical methods

All analyses were stratified by age group (0–5, 6–18, ≥ 18 years) to account for the pediatric and adult study arms of the ALLIANCE cohort and for phenotypic differences in children (preschool wheezers vs. asthmatics). HsCRP concentrations were log_10_-transformed before analysis. Group comparisons between preschool wheezers/asthmatics and HCs were performed using Fisher’s exact test for categorical variables and the Wilcoxon rank-sum test for continuous variables.

We obtained covariate-adjusted hsCRP values by fitting a linear mixed-effects model (lmer; lme4 R package) including BMI, gender, and square-root-transformed age as fixed effects, and a random intercept for study site (models were computed separately by age group and with disease status); model residuals were used as standardized log_10_ hsCRP values. Models with medication use as outcome were additionally standardized for GINA control status, which was included as a fixed effect. Adult models were additionally adjusted for smoking status (never, former, current smoking) and composite cardiovascular and inflammatory/respiratory comorbidity variables, referring to the previous 12 months as defined in the Supplementary Methods.

Differences in standardized log_10_ hsCRP levels were assessed using Student’s t-test for two-group comparisons, one-way analysis of variance (ANOVA) for multi-group comparisons and Pearson’s correlation coefficient for associations with continuous variables. Associations between hsCRP and clinical outcomes were examined with binary logistic regression for categorical outcomes and linear regression for continuous outcomes. Analyses of disease outcomes were restricted to cases (preschool wheezers and patients with asthma with an ASSESS score of 1 or higher). Odds ratios (ORs) and regression coefficients (β) with 95% confidence intervals (CIs) were reported for models including standardized log_10_-transformed hsCRP levels as predictor variables. For improved clinical interpretability, unadjusted descriptive hsCRP concentrations are additionally presented as median [IQR] in mg/L for key comparisons. A significance threshold of p ≤ 0.05 was applied. Given the exploratory nature of the study and the number of subgroup and outcome analyses performed, p-values were not adjusted for multiple testing. Results should therefore be interpreted as hypothesis-generating. All analyses were conducted in R version 4.3.2 [33].

## Results

### Demographic and clinical characteristics

**Fig. 1:**
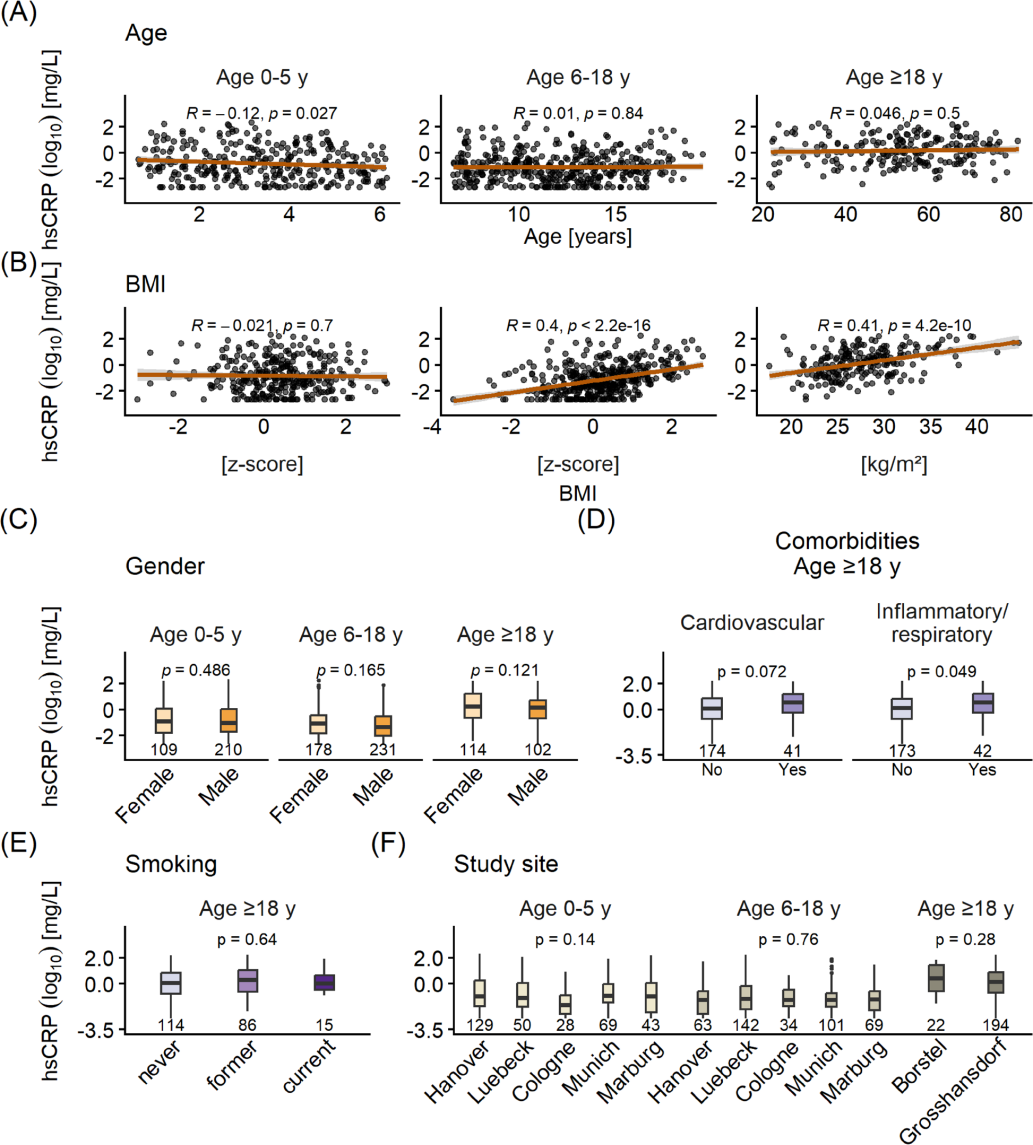
HsCRP in relation to key demographic and clinical determinants. Age-group specific differences between log_10_-transformed hsCRP concentrations and major covariates. Panels show associations of hsCRP with (**A**) age, **B** BMI, **C** sex, **D** adult comorbidities, namely cardiovascular (arterial hypertension, pulmonary hypertension, or other cardiopulmonary/cardiovascular comorbidities, including cardiovascular disease, arrhythmias, or peripheral arterial disease) and inflammatory/respiratory comorbidities (frequent respiratory tract infections, defined as ≥ 2 episodes per year, clinical signs and objective measurements strongly favoring a diagnosis of COPD, known alpha-1 antitrypsin deficiency, known bronchiectasis, or bronchiolitis obliterans organizing pneumonia (BOOP)), **E** smoking status, and **F** study site. *p*-values were calculated using Pearson’s correlation coefficient for continuous variables, Welch’s t-test for binary group comparisons, and one-way Analysis of Variance (ANOVA) for multi-group comparisons. Numbers below the boxplots indicate group sizes. BMI is presented as BMI z-score in children and as BMI in [kg/m^2^] in adults. According to WHO guidelines (see World Health organization, 2025), overweight was defined as 2 ≤ BMI-z < 3 for children (0-4y) and 1 ≤ BMI-z < 2 for children and adolescence (5-19y) and 25 ≤ BMI < 30 for adults; higher values were classified as obesity. hsCRP – high-sensitivity C-reactive protein. BMI – body mass index

A total of 944 study participants (age range: 0.5–79 years) with hsCRP measurements at the baseline visit were analyzed, stratified by age group. Demographic and clinical characteristics of HCs and patients are summarized in Table 1 and Supplementary Tables S2 and S3. HsCRP levels were negatively associated with age in early childhood (Fig. 1A) and were higher in participants aged ≥ 6 years with increased BMI (Fig. 1B). We observed no differences in hsCRP levels in relation to sex, smoking status and cardiovascular comorbidities in adults, or study site (Fig. 1C-F). Adults with inflammatory and respiratory comorbidities showed significantly higher hsCRP levels (Fig. 1D). We accounted for underlying variation in hsCRP levels related to age, sex, BMI, smoking status and comorbidities in adults, and study site by using covariate-standardized hsCRP values in subsequent analyses.

**Table 1:** Characteristics of study population across age groups

	**Healthy**	**Disease**	***p***
**Age 0-5y**	*n* = 62	*n* = 257	
Age [years]	3.00 [1.25;4.00]	3.00 [2.00;4.00]	0.792
Female	27 (43.5)	82 (31.9)	0.113
BMI (z-score)	0.16 [−0.59;0.84]	0.27 [−0.35;0.92]	0.405
Eosinophils [%]	2.70 [1.70;3.90]	3.55 [2.00;6.00]	**0.007**
Total IgE [kU/L]	21.3 [5.03;52.6]	38.7 [10.2;147]	**0.002**
Atopic	10 (16.4)	108 (42.7)	**< 0.001**
**Age 6-18y**	*n* = 204	*n* = 205	
Age [years]	11.0 [9.00;14.0]	11.0 [8.00;13.0]	0.07
Female	101 (49.5)	77 (37.6)	**0.019**
BMI (z-score)	−0.12 [−0.64;0.50]	0.16 [−0.42;0.91]	**0.002**
Eosinophils [%]	3.00 [1.72;4.85]	6.00 [3.20;10.0]	**< 0.001**
Total IgE [kU/L]	36.8 [14.4;123]	333 [125;585]	**< 0.001**
Atopic	69 (34.0)	175 (87.1)	**< 0.001**
FeNO [ppb]	9.95 [6.36;18.4]	17.3 [9.49;39.7]	**< 0.001**
**Age ≥ 18y**	*n* = 56	*n* = 160	
Age [years]	54.0 [38.2;63.2]	51.0 [43.0;63.2]	0.899
Female	24 (42.9)	90 (56.2)	0.116
BMI [kg/m^2^]	24.2 [22.3;26.8]	26.3 [24.1;29.1]	**0.002**
Eosinophils [%]	2.50 [2.00;4.00]	5.00 [2.00;8.00]	**< 0.001**
Total IgE [kU/L]	32.0 [12.0;54.8]	155 [50.7;410]	**< 0.001**
Atopic	17 (30.4)	101 (65.6)	**< 0.001**
FeNO [ppb]	15.5 [11.8;20.2]	25.0 [15.0;44.0]	**< 0.001**

Data are presented as n, n (%) or median (IQR), unless otherwise stated. Total n: *n* = 944. p-values are based on Fisher's exact and Wilcoxon rank sum test. *p*-values in bold indicate significant differences between groups (*p* ≤ 0.05). BMI: Body mass index. Atopy: measured with Euroimmun and/or Immuncap; any specific IgE ≥ 0.7 kU·L − 1. FeNO: Fractional Exhaled Nitric Oxide. IgE: Immunoglobolin E. Ppb: parts per billion

Missings: Eosinophils [%]: *n* = 27/944 (age < 6 *n* = 16; age ≥ 6 *n* = 10; age ≥ 18 *n* = 1); Total IgE [kU/L]: *n* = 99/944 (age < 6 *n* = 20; age ≥ 6 *n* = 54; age ≥ 18 *n* = 25); Atopic: *n* = 16/944 (age < 6 *n* = 5; age ≥ 6 *n* = 5; age ≥ 18 *n* = 6); FeNO [ppb]: *n* = 385/944 (age < 6 *n* = 291; age ≥ 6 *n* = 91; age ≥ 18 *n* = 3)

### HsCRP levels were increased in adult patients with asthma compared with healthy controls

**Fig. 2:**
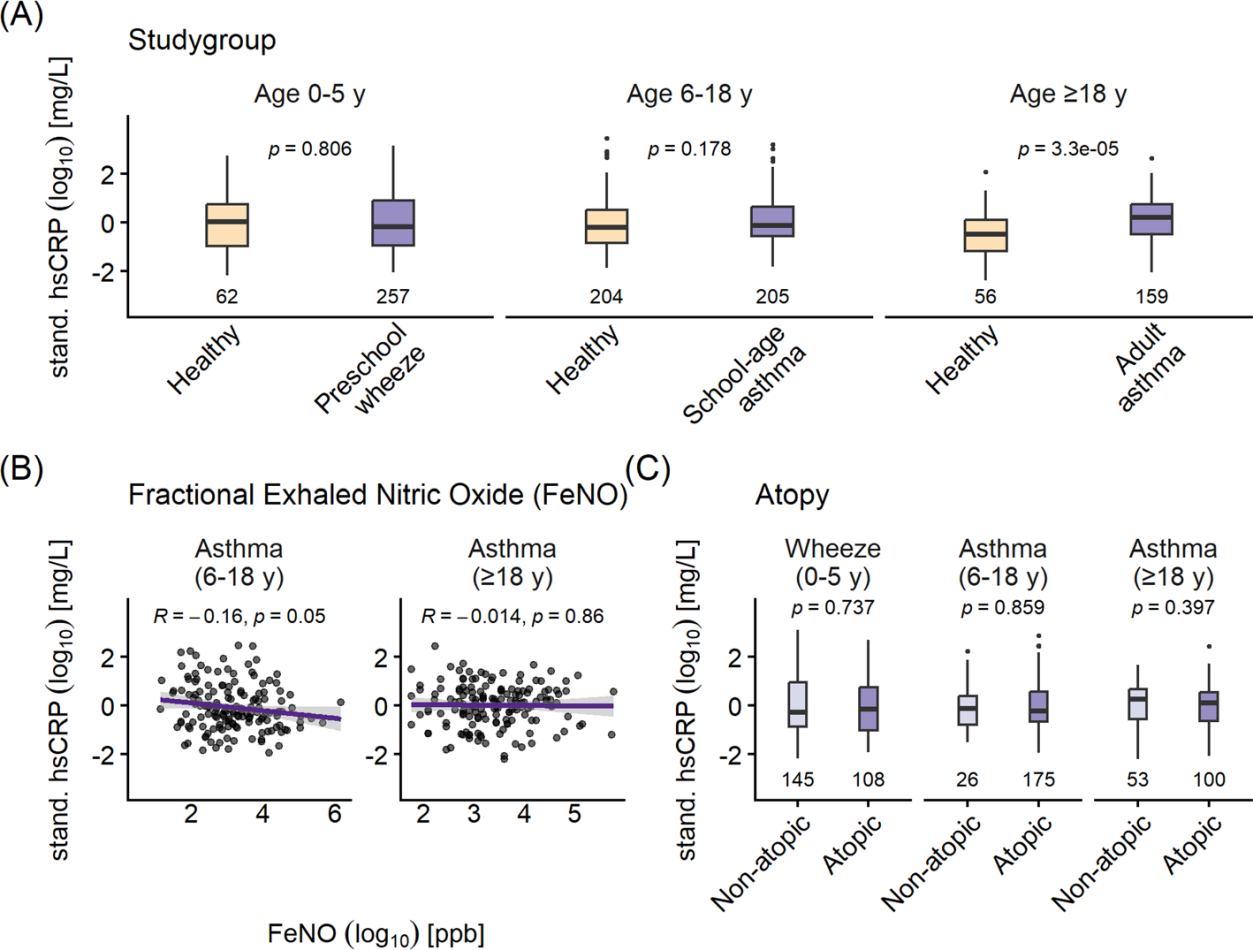
Comparison of hsCRP levels between healthy controls and patients. Boxplots show log_10_-transformed hsCRP concentrations standardized using linear mixed models including age, sex, BMI, and study site as covariates. In adult participants, smoking status and adult cardiovascular and inflammatory/respiratory comorbidities were additionally included as covariates. Standardized hsCRP concentrations are shown for **A** healthy individuals compared to preschool wheezers (age 0-5y) and asthmatics (6-18y and ≥ 18y), **B** patients in relation to FeNO [ppb] levels, **C** patients stratified by atopy status, defined as any specific IgE ≥ 0.7 kU L-1. *p*-values are derived by Welch’s t-test. Numbers below boxplots indicate group sizes. hsCRP – high-sensitivity C-reactive protein. IgE – Immunoglobulin E. FeNO – fractional exhaled nitric oxide

Figure 2A shows a comparison of hsCRP levels between HCs and patients, stratified by age group. In adults, asthma was associated with higher standardized hsCRP concentrations (OR = 2.17, 95% CI 1.52–3.18; median [IQR] hsCRP: HCs = 0.56 [0.29;1.04] mg/L; asthma = 1.58 [0.78;3.14] mg/L), whereas no clear association was observed in preschool children (OR = 1.03, 95% CI 0.82–1.29; median [IQR] hsCRP: HCs = 0.45 [0.18;0.84] mg/L; wheeze = 0.35 [0.17;1.14] mg/L) or school-age children (OR = 1.12, 95% CI 0.93–1.34; median [IQR] hsCRP: HCs = 0.25 [0.13;0.55] mg/L; asthma = 0.32 [0.16;0.74] mg/L).

There was a weak inverse association between hsCRP and fractional exhaled nitric oxide (FeNO) in school-age patients with asthma (Fig. 2B). No associations were found between hsCRP levels and Type-2 markers such as atopy (Fig. 2C), total IgE, and eosinophils (p ≥ 0.3; Supplementary Figure S2A-B) was found.

### HsCRP was associated with disease severity and chronic inflammation in patients with asthma

We further assessed the association between hsCRP levels and asthma severity outcomes, which are summarized across age groups in Table 2 (and Supplementary Table S4). Children had less severe asthma than adults, indicated by lower median ASSESS scores, better lung function measures (≥ 6 years) and a higher proportion of controlled asthma according to GINA.

**Table 2:** Asthma Severity Scoring System (ASSESS) and its components in patients across age groups

	**Wheeze (0-5y)**	**Asthma (6-18y)**	**Asthma (≥ 18y)**	***p***
Total	257	205	160	
ASSESS Score (1–20)		4.00 [3.00;6.00]	8.00 [5.00;12.0]	**< 0.001**
ACT Score^†^	22.0 [18.0;25.0]	22.0 [19.0;24.0]	20.0 [15.0;23.0]	**< 0.001**
FEV_1_ (z-score)		−0.59 [−1.23;0.10]	−1.76 [−2.70;−0.50]	**< 0.001**
FVC (z-score)		−0.13 [−0.72;0.69]	−0.38 [−1.03;0.36]	**0.019**
FEV_1_/FVC (z-score)		−0.76 [−1.61;−0.07]	−1.90 [−3.02;−0.97]	**< 0.001**
FEF 25–75 (z-score)		−0.91 [−1.79;−0.12]	−1.98 [−3.05;−0.86]	**< 0.001**
Inhaled steroid use^‡^	91 (52.9)	146 (91.2)	96 (87.3)	**< 0.001**
Systemic steroid use^‡^	85 (33.1)	42 (20.5)	28 (17.5)	**< 0.001**
Exacerbation^§^	21 (8.27)	10 (4.98)		0.231
Exacerbation^‡^	130 (50.6)	55 (26.8)	92 (57.5)	**< 0.001**
Hospitalisation^‡^	93 (36.2)	34 (16.6)	28 (17.5)	**< 0.001**
GINA control:				**< 0.001**
Controlled	99 (38.7)	96 (46.8)	39 (24.4)	
Partially controlled	89 (34.8)	81 (39.5)	60 (37.5)	
Uncontrolled	68 (26.6)	28 (13.7)	61 (38.1)	

Data are presented as n, n (%) or median (IQR), unless otherwise stated. Total n: *n* = 622. *p*-values are based on Fisher's exact and Wilcoxon rank sum test. *p*-values in bold indicate significant differences between groups (*p* ≤ 0.05)

ASSESS: Asthma Severity Scoring System, range from 0 (least severe) to 20 (most severe); patients with ASSESS score = 0 points were excluded. ACT: Asthma Control Test, range from 5 (poorest asthma control) to 25 (optimal asthma control); in children, the childhood ACT was used, range from 0 (poorest asthma control) to 27 (optimal asthma control). FEV_1_: forced expiratory volume in 1 s. FVC: forced vital capacity. FEF 25–75: forced expiratory flow at 25–75% of FVC. Exacerbation^§^: defined by GINA guidelines. Exacerbation^‡^: defined as use of systemic steroids and/or hospitalisation; systemic steroid use in adults defined as ≥ 3 consecutive days with oral corticosteroids (OCS) use or permanent OCS therapy. Hospitalisation: hospital visit due to respiratory symptoms. GINA control: Global Initiative for Asthma; guidelines from 2024

Missings: ASSESS Score (1–20): *n* = 257/622 (age < 6 *n* = 257); ACT Score: *n* = 174/622 (age < 6 *n* = 174); FEV_1_ (z-score): *n* = 261/622 (age < 6 *n* = 257; age ≥ 6 *n* = 4); FVC (z-score): *n* = 207/622 (age < 6 *n* = 203; age ≥ 6 *n* = 4); FEV_1_/FVC (z-score): *n* = 207/622 (age < 6 *n* = 203; age ≥ 6 *n* = 4); FEF 25–75 (z-score): *n* = 238/622 (age < 6 *n* = 208; age ≥ 6 *n* = 6; age ≥ 18 *n* = 24); Inhaled steroid use (12 months): *n* = 180/622 (age < 6 *n* = 85; age ≥ 6 *n* = 45; age ≥ 18 *n* = 50); Exacerbation (4 weeks): *n* = 167/622 (age < 6 *n* = 3; age ≥ 6 *n* = 4; age ≥ 18 *n* = 160); GINA control: *n* = 1/622 (age < 6 *n* = 1)

^†^In studygroup wheeze available only for age 4–5 years (y);

^‡^Previous 12 months prior to study visit;

^§^Previous 4 weeks prior to study visit

**Fig. 3:**
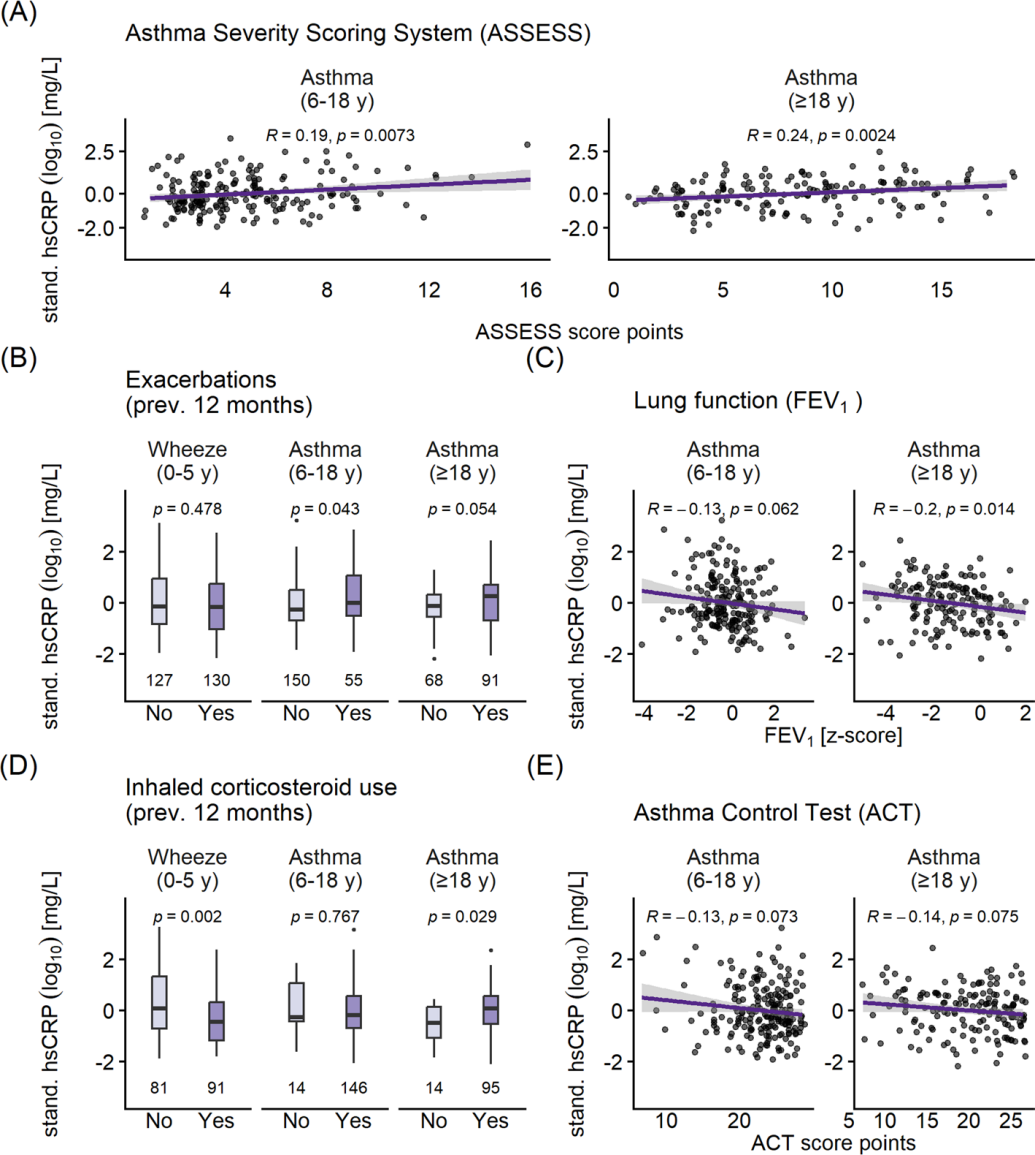
Association between ASSESS severity score components and hsCRP. Boxplots and scatterplots show log_10_-transformed hsCRP concentrations standardized for age, sex, BMI and study site. In adult participants, smoking status and adult cardiovascular and inflammatory/respiratory comorbidities were additionally included as covariates. For analysis of inhaled corticosteroid use, hsCRP concentrations were additionally standardized for GINA control status. Panels show associations with **A** ASSESS score points in patients aged ≥ 6 years, ranging from 1–20 (patients with zero points are excluded); **B** exacerbations, in children defined as hospitalisation due to symptoms of coughing and wheezing and/or systemic corticosteroid use within the previous 12 months and in adults defined as symptom worsening that requires oral corticosteroids (OCS) for at least three consecutive days, or application of permanent OCS therapy in the previous 12 months, **C** FEV_1_ in patients aged ≥ 6 years; **D** inhaled corticosteroid use within the previous 12 months (m) compared with no asthma medications, excluding patients receiving systemic corticosteroids and/or biologics; and **E** asthma symptom control quantified by Asthma Control Test (ACT) in patientsaged ≥ 6 years. *p*-values were calculated using Pearson’s correlation coefficient for continuous variables, and Welch’s t-test for binary comparisons. Numbers below the boxplots indicate group sizes. hsCRP – high-sensitivity C-reactive protein. ASSESS—Asthma Severity Scoring System. ACT – Asthma Control Test. FEV_1_—forced expiratory volume in 1 s

hsCRP levels were positively associated with ASSESS-defined severity (Fig. 3A, p < 0.01 in both age groups 6–18 and ≥ 18 years). School-age patients with asthma with exacerbations within the previous 12 months had increased hsCRP levels (Fig. 3B), whereas in adult patients with asthma, elevated hsCRP was associated with impaired lung function (FEV_1_, Fig. 3C). ICS use within the previous 12 months (Fig. 3D) was associated with increased standardized hsCRP concentrations in adult asthmatics (OR = 2.05, 95% CI 1.05–4.19; median [IQR] hsCRP: no ICS use = 0.72 [0.44;1.24] mg/L; ICS use = 1.64 [0.80;2.88] mg/L). In contrast, preschool wheezers with reported ICS use within the previous 12 months showed lower hsCRP levels (OR = 0.66, 95% CI 0.50–0.85; median [IQR] hsCRP: no ICS use = 0.54 [0.27;1.75] mg/L; ICS use = 0.25 [0.12;0.54] mg/L) (Fig. 3D). Asthma control quantified by ACT (Fig. 3E)or GINA control status (data not shown) was not associated with hsCRP.

### Elevated hsCRP was associated with recent exacerbations and ENT symptoms in preschool wheezers

**Fig. 4:**
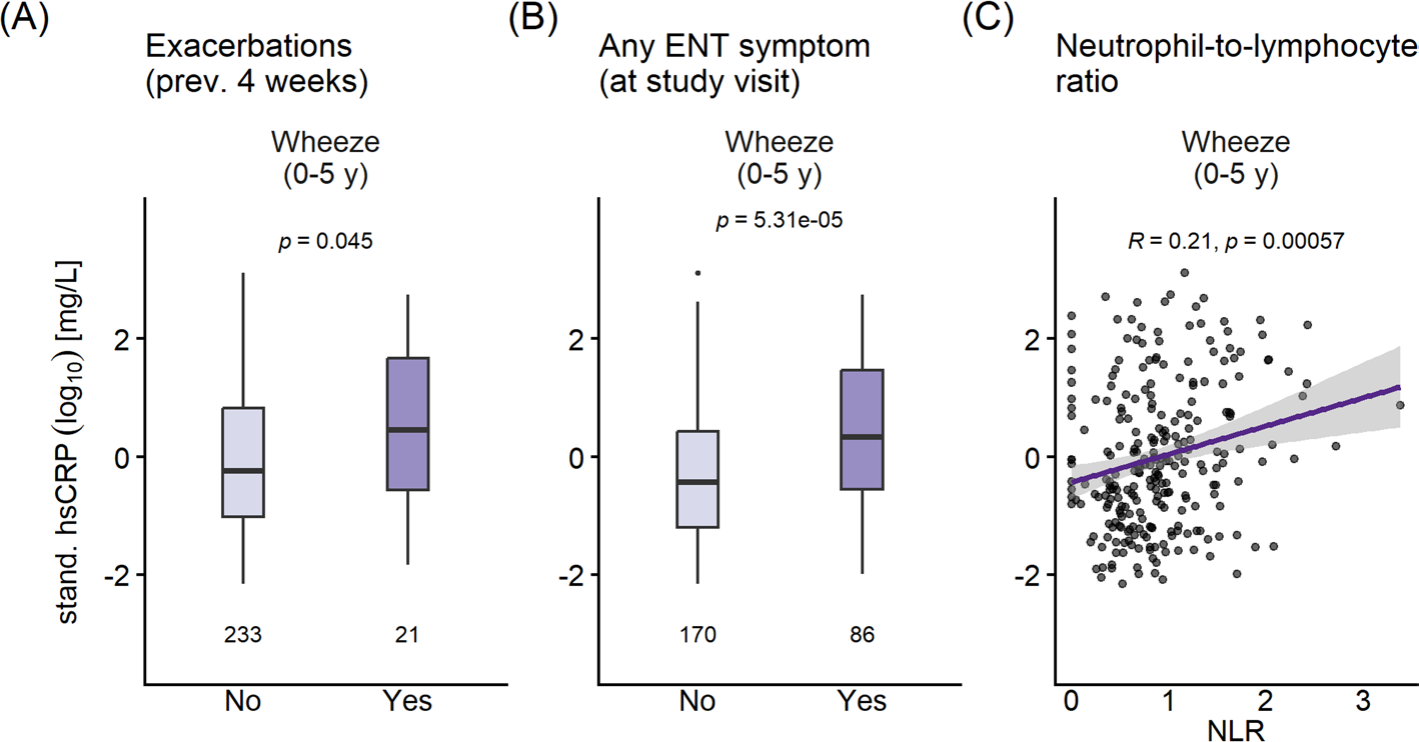
Differences in hsCRP levels in relation to recent exacerbations and prior infections in preschool wheezers. Associations of standardized hsCRP concentrations with recent exacerbations, ENT symptoms, and NLR in preschool wheezers. Boxplot and scatterplots show log_10_-transformed hsCRP concentrations standardized for age, sex, BMI and study site. Panels show associations with **A** recent exacerbations within the previous 4 weeks, defined for children according to Global Initiative for Asthma (GINA) guidelines for children; **B** any ENT symptom at study visit; and **C** neutrophilic-to-lymphocyte ratio (NLR). *p*-values were calculated using Pearson’s correlation coefficient for continuous variables, and Welch’s t-test for binary comparisons. Numbers below the boxplots indicate group sizes. hsCRP – high-sensitivity C-reactive protein. ENT – ear nose throat. NLR – neutrophil-to-lymphocyte ratio

To better understand the role of hsCRP in preschool wheezers, we conducted an additional analysis focusing on recent exacerbations, ENT symptoms suggestive of preceding infections, and the neutrophil-to-lymphocyte ratio (NLR) as a commonly used marker of an acute inflammatory response. Preschool wheezers with a GINA-defined exacerbation in the previous 4 weeks were more likely to have increased hsCRP levels than those without recent exacerbations (OR = 1.49, 95% CI 1.05–2.14; median [IQR] hsCRP: no exacerbation = 0.33 [0.17; 0.99] mg/L; exacerbation = 0.71 [0.33; 2.02] mg/L) (Fig. 4A). An elevation of hsCRP concentrations was also associated with ENT symptoms at the study visit (OR = 1.59, 95% CI 1.24–1.99; median [IQR] hsCRP: no ENT symptoms = 0.32 [0.14; 0.72] mg/L; ENT = 0.60 [0.28; 1.97] mg/L) (Fig. 4B). NLR was positively correlated with hsCRP levels (p < 0.001, Fig. 4C). School-age patients with asthma showed no differences in hsCRP levels in relation to recent exacerbations (Figure S3A) and ENT symptoms (Figure S3B).

## Discussion

This study demonstrated increased hsCRP in adult patients with asthma compared with HCs in the well-characterized all-age asthma cohort ALLIANCE and also in adults using inhaled corticosteroids. Conversely, preschool children showed lower hsCRP in association with ICS use, possibly reflecting its active anti-inflammatory effects early in life. Assessing this low-grade inflammation marker over the lifespan in our comprehensive longitudinal cohort of 944 participants provides the unique opportunity to evaluate its association with disease course and severity. In both childhood and adulthood, hsCRP was positively associated with asthma severity reflecting its relevance for the disease course and monitoring of clinical parameters.

Consistently from childhood to adulthood, elevated hsCRP levels were associated with a more severe asthma phenotype. This was represented by higher ASSESS scores. In particular, patients with asthmaand exacerbations, defined by systemic steroid use and/or event of hospitalisation within the previous 12 months showed higher hsCRP levels, as well as adult asthmatics with impaired poor lung function. Inhaled corticosteroid use was associated with decreased hsCRP in early life (0–5 years), whereas higher levels were identified in patients aged ≥ 18 years. In younger patients (0–5 years), increased hsCRP concentrations may reflect residual effects from a recently experienced exacerbation, accompanied by acute ENT symptoms that likely persisted as residual signs of a preceding infection.

### Study group effect between patients versus healthy controls

The wide age range of the ALLIANCE cohort revealed age-specific differences in hsCRP levels between individuals with wheeze/asthma and HCs. Compared with HCs, hsCRP levels were similar in preschool wheezers (0–5 years), but higher in school-age patients (6–18 years), and adult patients with asthma (≥ 18 years). After adjustment for confounders in the age group 6–18 years, the differences between patients and HCs were no longer observed.

The observed differences in hsCRP levels across the three age groups in our study may demonstrate age-related variation in underlying inflammatory processes. In our cohort, biomaterial was sampled in the stable disease phase. Similar hsCRP levels in HCs and preschool wheezers indicate no general elevation of systemic inflammation in preschool wheeze. The nature of preschool wheeze is clinically heterogeneous, including children with transient viral wheeze and children who may later develop persistent asthma. Yet, children with recent GINA-defined exacerbations and ENT symptoms at the study visit showed higher hsCRP levels. While acute systemic inflammation was minimized by excluding children with hsCRP ≥ 10 mg/L, body temperature ≥ 38.0 °C, or a neutrophil-to-lymphocyte ratio > 9, residual or resolving infection cannot be fully excluded. This is particularly relevant in preschool children, in whom frequent wheezing episodes are often triggered by infections. Symptoms, such as ENT symptoms may persist beyond the acute phase. Thus, the observed hsCRP elevation in this subgroup likely reflects infection-related or residual inflammatory activity rather than wheeze-specific systemic inflammation. This contrasts with a more persistent inflammatory profile of adult asthma, which may involve local airway inflammation and systemic responses may present with higher hsCRP levels even in the stable disease phase.

Our findings align with the assumption that disease manifestation and progression differ between pediatric and adult patients with asthma [34]. These differences could be reflected in systemic inflammation: in adults, results suggested a contribution of low-grade systemic inflammation to asthma [35]. Studies reported associations between hsCRP levels and respiratory symptoms [36], bronchial hyperresponsiveness [37], sputum eosinophils, and an inverse correlation with lung function indices [22, 38] in adult asthmatic patients [39, 40]. In contrast, the role of hsCRP on wheeze and asthma in children seems to be highly dependent on disease course: prior pediatric studies reported hsCRP elevations primarily during exacerbations. For instance, Zhu et al. observed higher CRP levels and an increased NLR in children (< 14 years) with asthma exacerbations [41]. Similarly, Soferman et al. reported elevated levels in children (2–12 years) during acute asthma exacerbations compared with remission periods [24].

BMI was positively associated with hsCRP in children aged ≥ 6 years and adults, in line with prior evidence identifying obesity as a major confounder of elevated hsCRP levels [42–45]. Adipose tissue-derived cytokines, such as IL-6, stimulate hepatic CRP synthesis and may therefore contribute to increased hsCRP concentrations irrespective of airway disease [46, 47]. Accordingly, all analyses were adjusted for BMI to account for its confounding effect.

Type-2 markers, such as atopy and eosinophils, were not associated with hsCRP in any age group. This contrasts with earlier reports suggesting hsCRP elevation predominantly in non-atopic asthma [36, 48]. However, the discrepancy may be explained by a different phenotype in ALLIANCE, with a predominance of atopic asthma in younger individuals. Our study highlights age-related differences in hsCRP levels between individuals with asthma and HCs, reflecting distinct inflammatory patterns across the lifespan.

### Asthma severity and hsCRP levels

This study identified an association between systemic inflammation and disease severity across all age groups, indicated by consistently elevated hsCRP levels. The higher burden of disease and inflammation was shown in patients with a higher severe ASSESS score.

In both school-age and adult asthmatics, severity of disease, as defined by the ASSESS score, was reflected by higher systemic inflammation in our study. Although the ASSESS score includes distinct dimensions of asthma severity (medication use, lung function, symptoms, and exacerbations), we recently showed in ALLIANCE that these dimensions do not necessarily show similar patterns across all patients and age groups [5].

HsCRP may indicate severity with systemic inflammation across these age groups in ALLIANCE, which is reflected by the association of systemic inflammation and declining lung function as described previously for adult asthmatics [37, 39, 49]. In our study, we also studied school-age children and observed an inverse, but not significant correlation between hsCRP and worsening of lung function. School-age patients with asthma with exacerbations within the last 12 months showed higher hsCRP. A mediating effect induced by asthma exacerbations appearing with bronchial hyperresponsiveness was also associated with elevated CRP [37].

In preschool wheezers, we identified an elevation of hsCRP levels in individuals shortly after GINA-defined exacerbations (within the previous 4 weeks). Similarly, a recent study emphasized the link between respiratory viruses and asthma exacerbations in children [50]. In ALLIANCE, 33% of preschool wheezers showed signs of ENT symptoms, which were associated with elevated hsCRP levels.

An association between hsCRP levels and ACT was not found. However, evidence remains inconclusive, with some studies demonstrating a relation [51] and others reporting no association [52]. This may be due to differences in study design and smaller study populations as well as inclusion of older age groups as compared to ALLIANCE and distinct phenotypes.

Importantly, our findings indicated that considering individual clinical features separately rather than patterns related to hsCRP might be insufficient. Systemic inflammation in disease severity may be attributable not only to certain clinical characteristics. The ASSESS score can be useful in determining factors associated with systemic inflammation.

Overall, our findings suggest that hsCRP reflects aspects of asthma-related disease burden across age groups, including severity-related features, exacerbations, and lung function impairment. The magnitude of several associations was modest. Therefore, the potential relevance of hsCRP may lie in complementing clinical and inflammatory phenotyping in wheeze and asthma patients, and particularly in adults. Further investigation will evaluate the potential of hsCRP as a measure of asthma severity, especially for exacerbations and inflammation-associated lung function decline in adult patients in our cohort in a longitudinal manner. Elevated hsCRP levels in preschool wheezers seems to be indicative of systemic inflammation associated with a recent exacerbation and/or infection.

### Treatment with inhaled corticosteroids

We identified age-specific differences in hsCRP levels related to inhaled corticosteroid use within the ALLIANCE cohort. In adults, higher hsCRP levels were associated with ICS therapy. ICS use and treatment intensity in adults may reflect more severe asthma phenotypes, associated with elevated hsCRP levels rather than independent treatment effects. This interpretation is in line with the finding that systemic inflammation is likely to be associated with more severe or treatment-resistant phenotypes [53–55]. A previous study linked higher doses of ICS and systemic steroids to elevated levels of hsCRP and IL-6 in patients with adult-onset asthma. The authors attributed this finding to a more severe, steroid-resistant asthma phenotype accompanied by systemic inflammation [45]. Despite treatment, systemic inflammation may also be increased due to a higher prevalence of comorbidities such as metabolic syndrome or cardiovascular disease [44, 48, 56].

In contrast, ICS therapy in children was associated with lower hsCRP concentrations. This observation is compatible with a potential anti-inflammatory effect of corticosteroid treatment, which inhibits the transcription of several inflammatory genes [57] and may thereby reduce hsCRP levels [22, 58]. This suggested link between ICS therapy and reduced inflammation supports the concept that anti-inflammatory therapy is effective, particularly in young children, and requires confirmation in longitudinal analyses. This contrast between age groups can only be disentangled in a cohort spanning several decades, such as the ALLIANCE cohort. Importantly, the finding was independent of asthma control status as we adjusted for GINA control status.

Of note, some facets should be noted. Based on some modest effects, the explanatory value for individual risk stratification is limited. Medication intake was recorded using questionnaires, and treatment adherence, especially in children and adolescents, is difficult to assess in large cohort studies. Thus, the observed effects might have been diluted suggesting an even stronger association between hsCRP and treatment. Residual confounding, as inherent to observational studies might have resulted in bias away from the null. However, the differential effects across age groups argue against overestimation of effects. Analyses were performed without formal adjustment for multiple testing; therefore, nominal p-values should be interpreted with caution and findings considered exploratory. Due to the cross-sectional design, no causal conclusions can be drawn. The challenge of the cross-sectional design will be addressed by future longitudinal analyses in the ALLIANCE cohort with its extensive follow-up.

## Conclusions

This study identified elevated hsCRP levels in adults with asthma and in patients with more severe disease-related outcomes, including higher ASSESS scores, lung function impairment, treatment exposure, and exacerbations. In adults, higher hsCRP levels in patients using ICS may reflect greater disease severity and treatment intensity rather than an independent corticosteroid effect. In school-age children, elevated hsCRP was associated with exacerbations within the previous year. In preschool wheezers, increased hsCRP levels were associated with recent infections and ENT symptoms. In this age group, lower hsCRP levels among ICS users were compatible with a potential anti-inflammatory effect, which needs further assessment while accounting for differences in disease severity, clinical management, and recent infections. These age-dependent effects may reflect varying disease courses across the lifespan and indicate progression of asthma in adults. The potential predictive and clinical value of hsCRP in asthma management will be evaluated in future longitudinal studies.

## Supplementary Information

Supplementary Material 1: Additional material including supplementary methods, legends of supplementary figures and supplementary tables.

Supplementary Material 2: Supplementary Figure S1.

Supplementary Material 3: Supplementary Figure S2.

Supplementary Material 4: Supplementary Figure S3.

## Data Availability

The datasets generated and analysed during the current study are not publicly available due to ethical restrictions but are available from the corresponding author on reasonable request.

## Abbreviations

ALLIANCE: All Age Asthma Cohort
ACT: Asthma Control Test
ASSESS: Asthma Severity Scoring System
BMI: Body mass index
COPD: Chronic obstructive pulmonary disease
CI: Confidence interval
CRP: C-reactive protein
DZL: German Centre of Lung Research
ENT: Ear, nose, throat
FEV_1_: Forced expiratory volume in 1 s
Fig: Figure
FeNO: Fractional Exhaled Nitric Oxide
FVC: Forced vital capacity
GINA: Global Initiative for Asthma
HC: Healthy controls
hsCRP: High-sensitivity C-reactive protein
ICS: Inhaled corticosteroids
KiGGS: German Health Interview and Examination Survey for Children and Adolescents
LABA: Long-acting β agonists
NLR: Neutrophil-to-lymphocyte ratio
OCS: Oral corticosteroids
OR: Odds ratio
p: *P*-value
