# Supplementary material for: Increased high sensitivity C-reactive protein in more severe wheeze/asthma phenotypes in child- and adulthood in ALLIANCE
Source: Respir Res. 2026 Jul 27;27:294. doi: 10.1186/s12931-026-03840-x (PMC13412301; doi:10.1186/s12931-026-03840-x)
Supplement: Supplementary file 1 — Supplementary Material 1: Additional material including supplementary methods, legends of supplementary figures and supplementary tables. [file 12931_2026_3840_MOESM1_ESM.pdf]

Supplement

Increased high sensitivity C-reactive protein in more severe wheeze/asthma phenotypes in child- and adulthood in ALLIANCE

Short running title: Elevated hsCRP in severe wheezer and asthmatics

Lena Lagally[1,2,3], Lena Ullemeyer[1,2], Jimmy Omony[2,4], Kristina Gottschau[1,2], Francesco Foppiano[1,3], Mustafa Abdo[5,6,7], Vera Veith[5,6], Clemens Thölken[8], Alexander Hose[1,2], Nicole Maison[1,2,4], Ruth Grychtol[9,10], Anna-Maria Dittrich[9,10,11], Lennart Riemann[9,10,12], Markus Weckmann[13,14,15], Inke König[14,16], Lea Kronziel[14,16], Sabina Illi[2,4], Silke van Koningsbruggen-Rietschel[17], Klaus F Rabe[5,6], Harald Renz[18,19], Gesine Hansen[9,10,11], Folke Brinkmann[13,14], Matthias V Kopp[13,14,20], E. von Mutius[1,2,4], Thomas Bahmer[5,6], Markus Ege[1,2,4], Chrysanthi Skevaki[18,19,21,22*], Bianca Schaub[1,2,23*], ALLIANCE Study Group

* shared last authorship.

1) Department of Paediatric Allergology, Dr. von Hauner Children's Hospital, LMU University Hospital, LMU Medizin, Ludwig-Maximilians-Universität München, Munich, Germany

2) Comprehensive Pneumology Center, Munich (CPC-M), German Center for Lung Research (DZL), Munich, Germany

3) Institute for Medical Information Processing, Biometry and Epidemiology (IBE), LMU Medicine, Ludwig-Maximilians-Universität München, Pettenkofer School of Public Health, Munich, Germany

4) Institute for Asthma and Allergy Prevention (IAP), Helmholtz Zentrum Munich, German Research Center for Environmental Health (GmbH), Neuherberg, Germany

5) LungenClinic Grosshansdorf GmbH, Grosshansdorf, Germany

6) Airway Research Center North (ARCN), German Center for Lung Research (DZL), Grosshansdorf, Germany

7) Department of Pneumology and Critical Care Medicine, Thoraxklinik at Heidelberg University Hospital, Translational Lung Research Center Heidelberg, Heidelberg, Germany

8) Center for Synthetic Microbiology (SYNMIKRO), Philipps-Universität Marburg, Marburg, Germany

9) Department of Paediatric Pneumology, Allergology and Neonatology, Hannover Medical School, Hannover, Germany

10) Biomedical Research in Endstage and Obstructive Lung Disease Hannover (BREATH), German Center for Lung Research (DZL), Hannover, Germany

11) Cluster of Excellence RESIST (EXC 2155), Hannover Medical School, Hannover, Germany

12) Institute of Immunology, Hannover Medical School, Hannover, Germany

13) University Children's Hospital, Luebeck, Germany

14) Airway Research Center North (ARCN), German Center for Lung Research (DZL), Luebeck, Germany

15) Division of Epigenetics in Chronic Lung Disease, Priority Area Chronic Lung Diseases, Leibniz Lung Center, Research Center Borstel, Borstel, Germany

16) Institute for Medical Biometry and Statistics, University Luebeck, University Medical Centre Schleswig-Holstein, Campus Luebeck, Luebeck, Germany

17) Department of Pediatrics, Faculty of Medicine and University Hospital Cologne, University of Cologne, Cologne, Germany

18) Institute of Laboratory Medicine and Pathobiochemistry, Molecular Diagnostics, University of Marburg, Marburg, Germany

19) University of Gießen and Marburg Lung Center (UGMLC); German Center for Lung Research (DZL), Gießen, Germany

20) Department of Paediatric Respiratory Medicine, Inselspital, University Children's Hospital of Bern, University of Bern, Bern, Switzerland

21) Department of Environmental Health, Harvard T.H. Chan School of Public Health, Boston, USA

22) Centre for Research and Education in Public Health (CEPHRE), Academy of Athens, Greece

23) German Center for Child and Adolescent Health (DZKJ), Dr. von Hauner Children's Hospital, LMU University Hospital, LMU Medizin, Ludwig-Maximilians-Universität München, Munich, Germany

Corresponding author

Bianca Schaub, LMU Munich, University Children's Hospital,

Lindwurmstrasse 4, D 80337, Munich, Germany,

Ph: +49-89-4400-5 7856.

E-mail: Bianca.Schaub@med.uni-muenchen.de

Supplemental figures

Figure S 1: Flow chart of study population selection. 8

Figure S 2: Relationship between hsCRP levels and Type-2 markers such as eosinophils, and total IgE across age groups. 8

Figure S 3: Differences in hsCRP levels in relation to recent exacerbations and prior infections in school-age asthmatics. 8

Supplemental tables

Table S 1: Calculation of the Asthma Severity Scoring System (ASSESS) score. 10

Table S 2: Supporting characteristics of pediatric study population 12

Table S 3: Supporting characteristics of the adult study population 14

Table S 4: Supporting clinical characteristics of patients across age groups 15

Supplemental methods

The study design and procedures of the All Age Asthma Cohort (ALLIANCE) are described in more detail elsewhere[1,2]. This section provides additional information and definitions about the included variables (dataset versions: children V9, adults 2024).

Asthma control

Asthma symptom control was defined for each patient by GINA guidelines[3] according to the categories, controlled, partially controlled, and uncontrolled. In addition, severity in adults was classified as mild, moderate and severe according to GINA treatment steps guidelines[3].

Medication intake

Apart from steroid use, the intake of long-acting muscarinic antagonist (LAMA), Tiotropium, and Omalizumab within the last 12 months prior to study visit was assessed (pediatric samples: only one patient with LAMA use; Tiotropium use was not reported). For pediatric patients, recent medication intake (last 4 wks) was also reported.

Atopy

Atopy was defined as any specific IgE ≥0.7 kU·L−1 against at least one of 36 aero- or food allergens, measured by Euroline (Euroimmun, Germany) and/or ImmunoCAP IgE assays (Thermo Fisher Scientific/Phadia, Uppsala, Sweden).

Lung function

Lung function was determined using a spirometer or body plethysmograph. Forced expiratory volume in 1 second (FEV_1), forced vital capacity (FVC) and FeNO (ppb) were measured by physicians according to the European Respiratory Society standards[4]. Using GLI-2012 equations[5] (Global Lung Function Initiative), z-scores were computed for FEV_1, FVC, FEV_1/FVC and FEF_{25-75}.

Body mass index

In children, body mass index (BMI) z-scores were computed using German Health Interview and Examination Survey for Children and Adolescents (KiGGS) percentiles for anthropometric measures[6]. In adults, BMI was calculated as weight in kilograms divided by the square of height in meters. According to WHO guidelines[7], overweight was defined as 2≤BMI-z<3 for children (0-4y) and 1≤BMI-z<2 for children and adolescence (5-19y) and 25≤BMI<30 for adults; higher values were classified as obesity.

Neutrophil-to-lymphocyte ratio (NLR)

The neutrophil-to-lymphocyte ratio was calculated by dividing the percentage of neutrophils and lymphocytes in a complete blood count analysis.

Adult comorbidities

Adult comorbidities were assessed using binary questionnaire items asked by the physician (no/yes). For the present analyses, two composite comorbidity variables were derived, referring to the previous 12 months.

The cardiovascular comorbidity variable was defined as positive if at least one of the following conditions was reported: arterial hypertension, pulmonary hypertension, or other cardiopulmonary/cardiovascular, including cardiovascular disease, arrhythmias, or peripheral arterial disease. Participants were classified as negative if all available items were coded as no. The inflammatory/respiratory comorbidity variable was defined as positive if at least one of the following conditions was reported: frequent respiratory tract infections, defined as ≥2 episodes per year, clinical signs and objective measurements strongly favoring a diagnosis of COPD, known alpha-1 antitrypsin deficiency, known bronchiectasis, or bronchiolitis obliterans organizing pneumonia (BOOP).

Supplemental references

1. Fuchs O, Bahmer T, Weckmann M, et al. The all age asthma cohort (ALLIANCE) - from early beginnings to chronic disease: a longitudinal cohort study. *BMC Pulm Med*. 2018;18(1):140. doi:10.1186/s12890-018-0705-6

2. Maison N, Omony J, Illi S, et al. T2-high asthma phenotypes across lifespan. *Eur Respir J*. 2022;60(3). doi:10.1183/13993003.02288-2021

3. 2025 GINA Main Report. Global Initiative for Asthma - GINA. Accessed August 4, 2025. https://ginasthma.org/2025-gina-strategy-report/

4. Louis R, Satia I, Ojanguren I, et al. European Respiratory Society guidelines for the diagnosis of asthma in adults. *Eur Respir J*. 2022;60(3). doi:10.1183/13993003.01585-2021

5. Quanjer PH, Stanojevic S, Cole TJ, et al. Multi-ethnic reference values for spirometry for the 3–95-yr age range: the global lung function 2012 equations. *Eur Respir J*. 2012;40(6):1324-1343. doi:10.1183/09031936.00080312

6. Federal Health Reporting. *Reference Percentiles for Anthropometric Measures and Blood Pressure Based on the German Health Interview and Examination Survey for Children and Adolescents 2003–2006 (KiGGS)*.; 2016. https://edoc.rki.de/bitstream/handle/176904/3271/23s3ntHQtKbus.pdf?sequence=1

7. World Health Organization. *Obesity and Overweight*. WHO; 2025. Accessed July 31, 2025. https://www.who.int/news-room/fact-sheets/detail/obesity-and-overweight

8. Skevaki C, Tafo P, Eiringhaus K, et al. Allergen extract- and component-based diagnostics in children of the ALLIANCE asthma cohort. *Clin Exp Allergy J Br Soc Allergy Clin Immunol*. 2021;51(10):1331-1345. doi:10.1111/cea.13964

Supplemental figure legends

Figure S 1: Flow chart of study population selection.

Flow chart of study population selection for pediatric (Kinder Register Asthma Study - KIRA) and adult (Erwachsene Register Asthma Study - ERA) arm of the All Age Asthma Cohort (ALLIANCE). Exclusion criteria and the number of excluded samples are shown in a grey box. hsCRP – high-sensitivity C-reactive protein. NLR – Neutrophil-Lymphocyte Ratio. BMI – body mass index. ASSESS – Asthma Severity Scoring System.

Figure S 2: Relationship between hsCRP levels and Type-2 markers such as eosinophils, and total IgE across age groups.

Age-group specific differences of standardized hsCRP concentrations with eosinophils and total IgE. Scatterplots show log_{10}-transformed hsCRP concentrations standardized for age, sex, BMI and study site. In adult participants, smoking status and adult cardiovascular and inflammatory/respiratory comorbidities were additionally included as covariates. Panels show associations with (A) eosinophils [%], and (B) total IgE [kU/L]. *p*-values were calculated using Pearson's correlation coefficient for continuous variables. hsCRP – high-sensitivity C-reactive protein. IgE – Immunoglobulin E.

Figure S 3: Differences in hsCRP levels in relation to recent exacerbations and prior infections in school-age asthmatics.

Associations of standardized hsCRP concentrations with recent exacerbations and ENT symptoms in school-age asthmatics. Boxplots show log_{10}-transformed hsCRP concentrations standardized for age, sex, BMI and study site. In adult participants, smoking status and adult cardiovascular and inflammatory/respiratory comorbidities were additionally included as covariates. Panels show associations with (A) recent exacerbations within the previous 4 weeks, defined according to Global Initiative for Asthma (GINA) guidelines for children; (B) any ENT symptom at study visit. *p*-values were calculated using Welch's t-test for binary

comparisons. Numbers below the boxplots indicate group sizes. hsCRP – high-sensitivity C-reactive protein. ENT – ear nose throat.

Supplemental tables

Table S 1: Calculation of the Asthma Severity Scoring System (ASSESS) score.

Component	Weighting	ASSESS score
ACT score	23-25 points (in children aged 6-11 y: 23-27 points*)	0 point
	20-22 points (in children aged 6-11 y: 20-23 points*)	1 point
	17-19 points	2 points
	14-16 points	3 points
	11-13 points	4 points
	8-10 points	5 points
	5-7 points	6 points
Lung function (FEV_1% predicted)	≥80% predicted	0 point
	70%-80% predicted	1 point
	60%-70% predicted	2 points
	<60% predicted	3 points
Asthma treatment† (current medication)	No treatment	0 point
	Albuterol only	1 point
	Low-dose‡ ICS only or LTRA only	2 points
	Low-dose ICS and at least 1 controller§ or medium-dose‡ ICS only or high-dose‡ ICS only	3 points
	Medium-dose ICS and at least 1 controller or high-dose ICS and at least 1 controller	4 points
	High dose ICS and at least 2 controllers	5 points
	Systemic corticosteroids	1 point
	Current biological	1 point
Exacerbations	Prednisone burst	2 points
	Prednisone burst + hospitalization‖	4 points

The calculation of the Asthma Severity Scoring System (ASSESS) score has already been validated and published in the ALLIANCE cohort (Grychtol et al, 2023). The table has been reconstructed from this publication.

The table lists the 4 components of the ASSESS score and their weighting. The final score is calculated by summing up the points from each component to a maximum score of 20, with higher scores indicating increased asthma severity.
ICS, Inhaled corticosteroid; LABA, long-acting b-agonist; LAMA, long-acting muscarinic antagonist; LTRA, leukotriene receptor antagonist.
*In children aged 6 to 11 years, the Childhood ACT was used.
†In ALLIANCE, ‘‘recent’’ medication use (i.e., use in the past 4 wks.) was documented for each study visit.
‡Low-, middle-, and high-dose ICSs were defined according to the GINA guidelines using age-dependent and substance-specific cutoffs.
§Controllers are LTRA, LABA, LAMA, and theophylline (for step 5, also low-dose oral corticosteroids). Pediatric patients received only LABA and LRTA.
‖Prednisone bursts and hospitalizations refer to the past 12 months.

4

Table S 2: Supporting characteristics of pediatric study population

	Age 0-5 yrs			Age 6-18 yrs		
	**Healthy**	**Disease**	***p***	**Healthy**	**Disease**	***p***
Total	n=62	n=257		n=204	n=205	
hsCRP [mg/L]	0.45 [0.18;0.84]	0.35 [0.17;1.14]	0.804	0.25 [0.13;0.55]	0.32 [0.16;0.74]	**0.044**
Neutrophil counts [%]	41.0 [30.5;49.7]	39.0 [31.2;46.1]	0.706	48.5 [43.0;54.0]	46.1 [39.9;51.0]	**0.002**
Leucocyte counts [%]	8.68 [6.72;10.1]	8.66 [7.27;10.7]	0.208	6.55 [5.60;7.75]	7.34 [6.22;8.60]	**<0.001**
Lymphocyte counts [%]	47.7 [38.9;57.6]	46.2 [38.3;55.0]	0.643	38.0 [33.0;44.0]	38.0 [33.0;43.0]	0.932
Eczema:	2 (3.33)	62 (24.3)	**0.001**	14 (6.90)	86 (42.4)	**<0.001**
Rhinoconjunctivitis:	0 (0.00)	27 (10.6)	**0.018**	9 (4.43)	100 (49.5)	**<0.001**
Smoking exposure:	12 (19.4)	79 (30.7)	0.104	45 (22.8)	65 (31.9)	0.056
Maternal history of asthma:	12 (19.4)	55 (21.7)	0.812	22 (10.9)	53 (26.6)	**<0.001**
Paternal history of asthma:	3 (4.84)	53 (20.9)	**0.005**	17 (8.46)	39 (19.6)	**0.002**
Maternal history of rhinoconjunctivitis:	14 (23.3)	81 (31.9)	0.254	63 (31.3)	73 (36.9)	0.290
Paternal history of rhinoconjunctivitis:	21 (35.0)	88 (34.6)	1.000	57 (28.4)	69 (34.8)	0.198
Education mother:			**0.011**			**<0.001**
high	44 (72.1)	137 (53.7)		122 (60.7)	88 (43.1)	
low	1 (1.64)	29 (11.4)		14 (6.97)	38 (18.6)	
middle	16 (26.2)	89 (34.9)		65 (32.3)	78 (38.2)	
Education father:			**<0.001**			**<0.001**
high	48 (80.0)	128 (52.0)		125 (64.1)	83 (43.5)	
low	2 (3.33)	39 (15.9)		28 (14.4)	51 (26.7)	
middle	10 (16.7)	79 (32.1)		42 (21.5)	57 (29.8)	
Siblings:	42 (67.7)	170 (66.1)	0.929	168 (82.4)	163 (79.5)	0.545
Study center:			**<0.001**			0.146
Cologne	6 (9.68)	22 (8.56)		14 (6.86)	20 (9.76)	
Hannover	24 (38.7)	105 (40.9)		39 (19.1)	24 (11.7)	
Lübeck	3 (4.84)	47 (18.3)		64 (31.4)	78 (38.0)	
Marburg	4 (6.45)	39 (15.2)		38 (18.6)	31 (15.1)	
Munich	25 (40.3)	44 (17.1)		49 (24.0)	52 (25.4)	

Note: Data are presented as n, n (%) or median (IQR), unless otherwise stated. p-values are based on Fisher's exact and Wilcoxon rank sum test. *p*-values in bold indicate significant differences between groups ($p \leq 0.05$).

Eczema: diagnosed by doctor (ever). Rhinoconjunctivitis: diagnosed by doctor (ever). hsCRP – high-sensitivity C-reactive protein.
Missings: Neutrophil counts: n=23/728 (age<6 n=18; age≥6 n=5); Leucocyte counts: n=13/728 (age<6 n=8; age≥6 n=5); Lymphocyte counts: n=14/728 (age<6 n=8; age≥6 n=6); Eczema: n=7/728 (age<6 n=4; age≥6 n=3); Rhinoconjunctivitis: n=9/728 (age<6 n=5; age≥6 n=4); Smoking exposure: n=8/728 (age<6 n=0; age≥6 n=8); Asthma (mother): n=13/728 (age<6 n=4; age≥6 n=9); Asthma (father): n=13/728 (age<6 n=4; age≥6 n=9); Rhinoconjunctivitis (mother): n=15/728 (age<6 n=5; age≥6 n=10); Rhinoconjunctivitis (father): n=15/728 (age<6 n=5; age≥6 n=10); Education (mother): n=7/728 (age<6 n=3; age≥6 n=4); Education (father): n=36/728 (age<6 n=13; age≥6 n=23).

Table S 3: Supporting characteristics of the adult study population

	Healthy	Disease	*p*
Total	n=56	n=160	
hsCRP [mg/L]	0.56 [0.29;1.04]	1.61 [0.78;3.31]	**<0.001**
Neutrophil counts [%]	57.0 [49.0;60.2]	60.0 [53.0;66.0]	**0.001**
Leucocyte counts [%]	5.80 [5.11;6.61]	7.15 [5.86;8.54]	**<0.001**
Lymphocyte counts [%]	32.0 [26.0;37.0]	25.0 [20.5;30.5]	**<0.001**
Allergic asthma:			**<0.001**
allergic	16 (28.6)	110 (68.8)	
non-allergic	27 (48.2)	41 (25.6)	
unclear/mixed	13 (23.2)	9 (5.6)	
Eczema:	1 (1.79)	44 (27.5)	**<0.001**
Rhinoconjunctivitis:	15 (26.8)	99 (61.9)	**<0.001**
Smoking:			0.293
never	31 (55.4)	84 (52.5)	
current	6 (10.7)	9 (5.62)	
former	19 (33.9)	67 (41.9)	
Selected Comorbidities			
Cardiovascular	9 (16.1)	32 (20.1)	0.559
Inflammatory/respiratory	0 (0.00)	42 (26.4)	**<0.001**
Maternal history of asthma:	56 (100)	143 (89.4)	**0.008**
Paternal history of asthma:	52 (92.9)	144 (90.0)	0.714
Study center:			
Borstel	0 (0.00)	22 (13.8)	**0.008**
Grosshansdorf	56 (100)	138 (86.2)	

Note: Data are presented as n, n (%) or median (IQR), unless otherwise stated. p-values are based on Fisher's exact and Wilcoxon rank sum test. *p*-values in bold indicate significant differences between groups ($p \leq 0.05$).

Allergic asthma: for adult patients, allergic = asthmatic symptoms or symptoms of concomitant allergic rhinitis/conjunctivitis are triggered by aeroallergens; non-allergic = Patient History had to be supported by positive Skin-Prick results as indicated in the patient questionnaire. Pos. Prick-Test results without related symptoms were classified as unclear/ mixed. unclear/mixed = In Healthy Controls, this item indicates whether there is an atopic comorbidity is present, or not (i.e., allergic rhinoconjunctivitis); In Healthy controls, the box 'unclear/mixed' indicated individuals with positive Prick-test results but negative personal history with respect to seasonal rhino-conjunctivitis or asthma. Eczema: any symptoms of allergic dermatitis (ever). Rhinoconjunctivitis: any symptoms of rhinoconjunctivitis (ever). Smoking: never, current or former smoker. Selected comorbidities: we derived two composite variables for comorbidities, referring to the previous 12 months; one for cardiovascular comorbidities, indicating the presence of arterial hypertension, pulmonary hypertension, or other cardiopulmonary/cardiovascular comorbidities (e. g. cardiovascular disease, arrhythmias, peripheral artery disease); and one for inflammatory/respiratory comorbidities, based on frequent respiratory tract infections, clinical and objective measurements that favor a diagnosis of COPD, alpha-1 antitrypsin deficiency, bronchiectasis, and bronchiolitis obliterans. hsCRP – high-sensitivity C-reactive protein.
Missings: Neutrophil counts: for n=1/216 adults. Leucocyte counts: for n=1/216 adults. Lymphocyte counts: for n=1/216 adults. Comorbidities: for n=1/216 adults.

Table S 4: Supporting clinical characteristics of patients across age groups

	Wheeze (0-5y)	Asthma (6-18y)	Asthma (≥18y)	p
Total	n=257	n=205	n=160	
Age of first symptoms:				.
0-4 years	252 (98.1)	139 (69.2)	17 (11.0)	
4-6 years	0 (0.00)	0 (0.00)	9 (5.81)	
5-6 years	5 (1.95)	38 (18.9)	0 (0.00)	
6+ years	0 (0.00)	24 (11.9)	129 (83.2)	
Asthma diagnosis:	89 (35.2)	201 (99.5)	159 (99.4)	**<0.001**
GINA treatment steps:				.
mild	.	.	38 (23.8)	
moderate	.	.	59 (36.9)	
severe	.	.	63 (39.4)	
ICS use†:	85 (33.1)	72 (35.1)	.	0.717
ICS use‡:	134 (52.1)	95 (46.3)	43 (26.9)	**<0.001**
LABA/ICS use†:	30 (11.7)	106 (51.7)	.	**<0.001**
LABA/ICS use‡:	46 (17.9)	118 (57.6)	120 (75.0)	**<0.001**
LAMA use†:	0 (0.00)	1 (0.49)	.	0.444
LAMA use‡:	0 (0.00)	1 (0.49)	37 (23.1)	**<0.001**
Omalizumab use†:	0 (0.00)	7 (3.41)	.	**0.003**
Omalizumab use‡:	0 (0.00)	8 (3.90)	10 (6.25)	**<0.001**
Tiotropium use‡:	.	.	30 (18.8)	.

Note: Data are presented as n, n (%) or median (IQR), unless otherwise stated. p-values are based on Fisher's exact and Wilcoxon rank sum test. *p*-values in bold indicate significant differences between groups ($p \leq 0.05$).

† previous 4 wks prior to study visit.
‡ previous 12 months prior to study visit.
Asthma diagnosis: at baseline visit (ever). GINA: Global Initiative for Asthma. ICS: inhaled corticosteroid use. LABA: long-acting beta2-agonists. LAMA: Long-acting muscarinic antagonists.
Missings: Age of first symptoms: n=9/622 (age≥6 n=4; age≥18 n=5); Asthma diagnosis: n=7/622 (age<6 n=4; age≥6 n=3); GINA treatment steps: n=462/622 (age<6 n=257; age≥6 n=205); ICS (4 wks): n=160/622 (age≥18 n=160); LABA/ICS (4 wks): n=160/622 (age≥18 n=160); LAMA (4 wks): n=160/622 (age≥18 n=160); Omalizumab (4 wks): n=160/622 (age≥18 n=160); Tiotropium (12 months): n=462/622 (age<6 n=257; age≥6 n=205).
