# Supplementary figures and images for: Increased high sensitivity C-reactive protein in more severe wheeze/asthma phenotypes in child- and adulthood in ALLIANCE
Source: Respir Res. 2026 Jul 27;27:294. doi: 10.1186/s12931-026-03840-x (PMC13412301; doi:10.1186/s12931-026-03840-x)

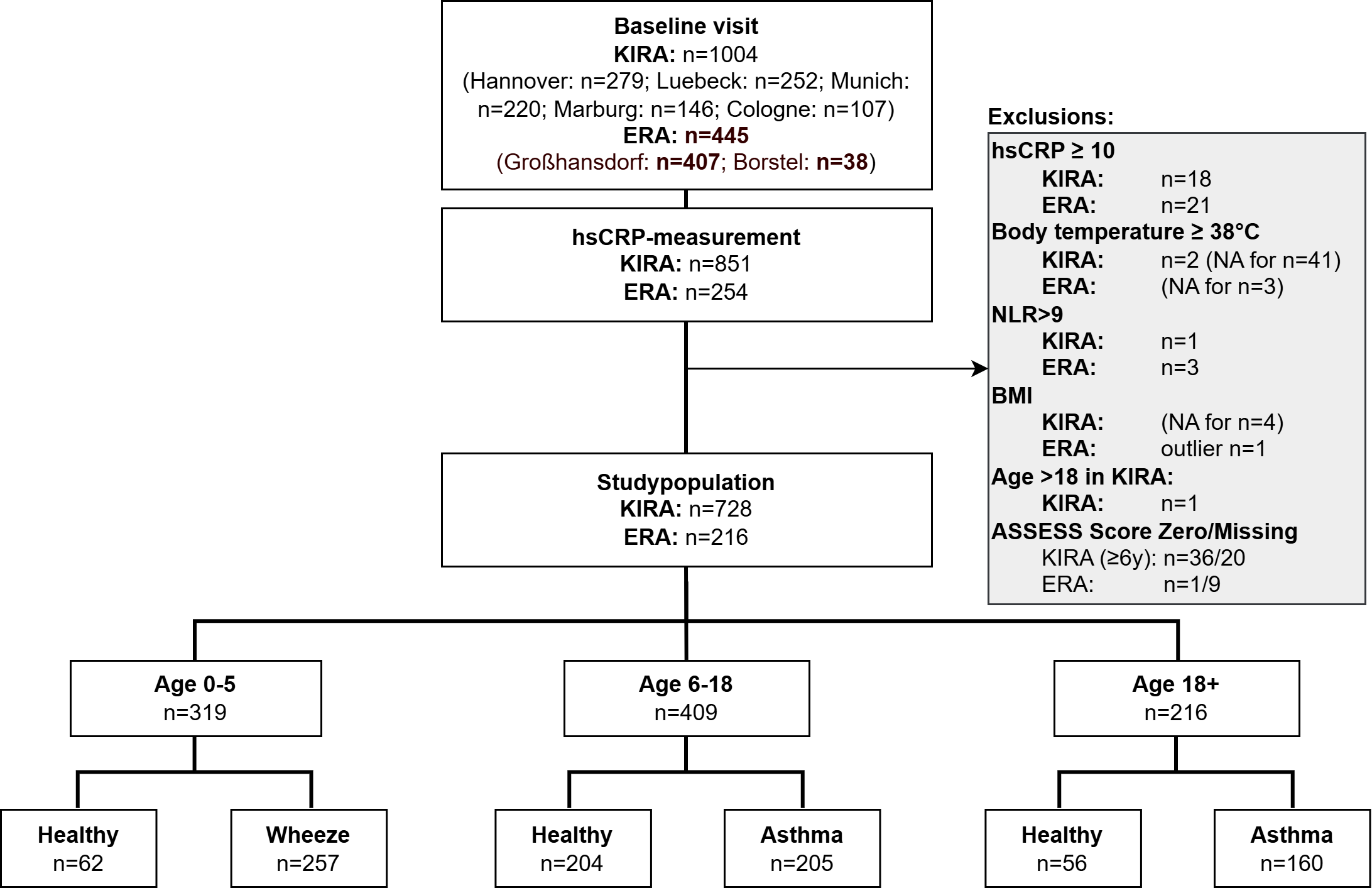

Supplement: Supplementary file 2 — Supplementary Material 2: Supplementary Figure S1. [file 12931_2026_3840_MOESM2_ESM.png]

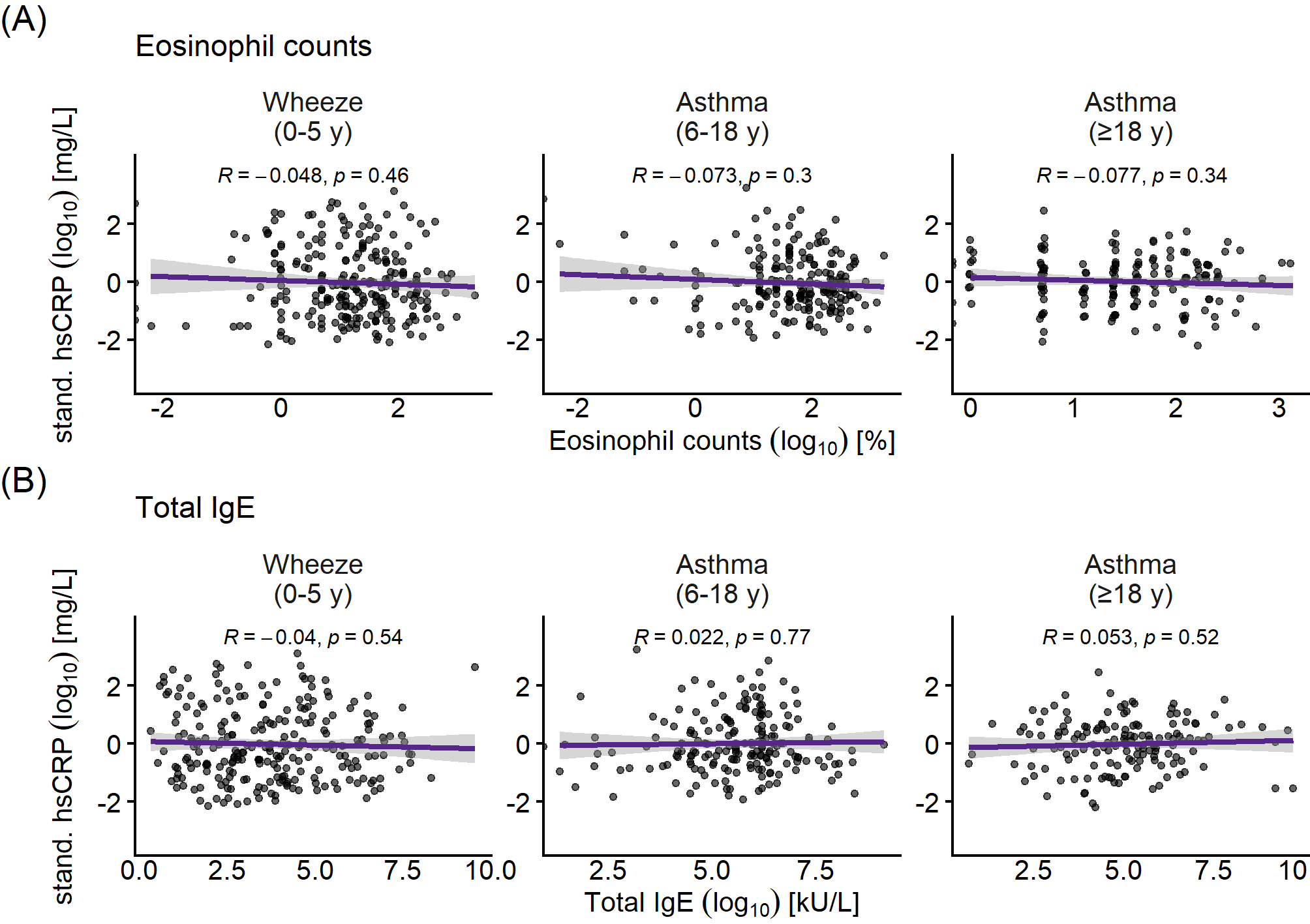

Supplement: Supplementary file 3 — Supplementary Material 3: Supplementary Figure S2. [file 12931_2026_3840_MOESM3_ESM.png]

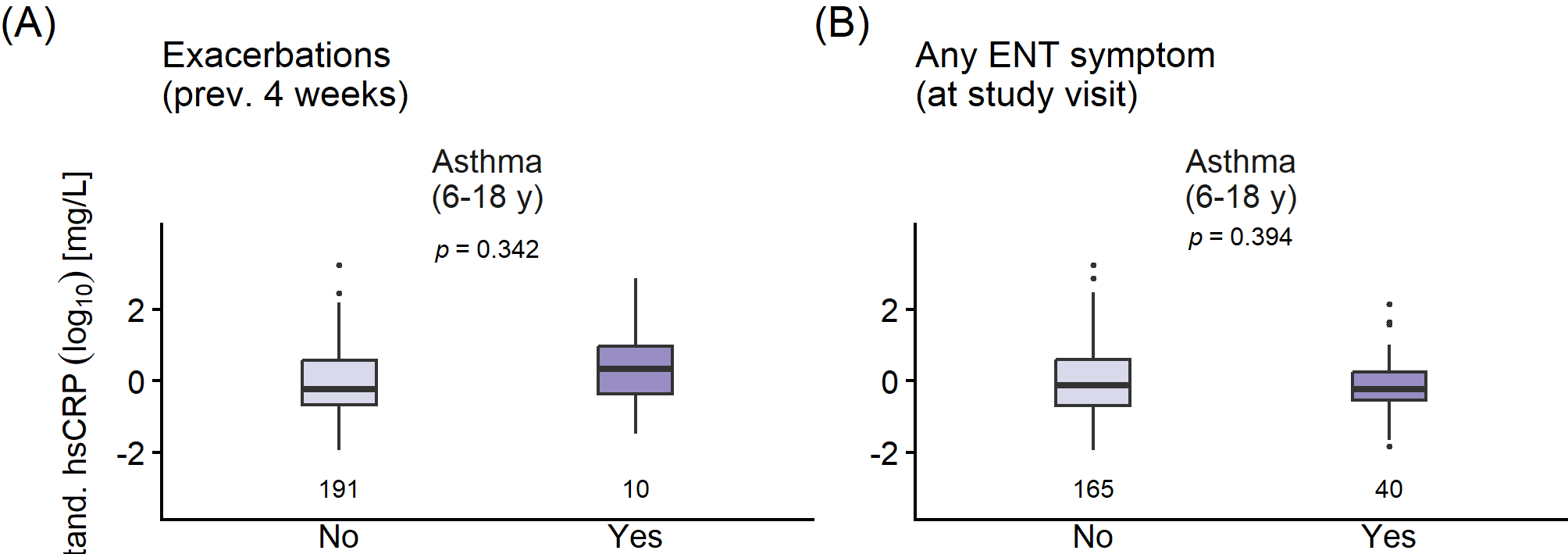

Supplement: Supplementary file 4 — Supplementary Material 4: Supplementary Figure S3. [file 12931_2026_3840_MOESM4_ESM.png]
